# The Role of Attention in Ambiguous Reversals of Structure-From-Motion

**DOI:** 10.1371/journal.pone.0037734

**Published:** 2012-05-22

**Authors:** Solveiga Stonkute, Jochen Braun, Alexander Pastukhov

**Affiliations:** Cognitive Biology Lab, Institute of Biology, Otto-von-Guericke Universität, Magdeburg, Germany; CNRS–Université Claude Bernard Lyon 1, France

## Abstract

Multiple dots moving independently back and forth on a flat screen induce a compelling illusion of a sphere rotating in depth (structure-from-motion). If all dots simultaneously reverse their direction of motion, two perceptual outcomes are possible: either the illusory rotation reverses as well (and the illusory depth of each dot is maintained), or the illusory rotation is maintained (but the illusory depth of each dot reverses). We investigated the role of attention in these ambiguous reversals. Greater availability of attention – as manipulated with a concurrent task or inferred from eye movement statistics – shifted the balance in favor of reversing illusory rotation (rather than depth). On the other hand, volitional control over illusory reversals was limited and did not depend on tracking individual dots during the direction reversal. Finally, display properties strongly influenced ambiguous reversals. Any asymmetries between ‘front’ and ‘back’ surfaces – created either on purpose by coloring or accidentally by random dot placement – also shifted the balance in favor of reversing illusory rotation (rather than depth). We conclude that the outcome of ambiguous reversals depends on attention, specifically on attention to the illusory sphere and its surface irregularities, but not on attentive tracking of individual surface dots.

## Introduction

**Figure 1:**
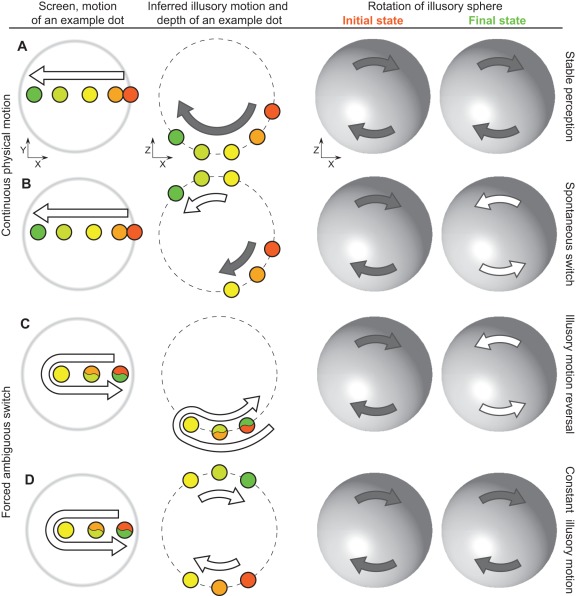
Structure-from-motion display and FAS paradigm. Left column: a 2D motion of an example dot on the screen. Middle column: inferred illusory depth and illusory rotation of an example dot, as if viewed from above. Right column: initial and final direction of illusory rotation of an illusory sphere, as if viewed from above. Colour denotes position of the dot at various times: red – initial location, green – final location, intermediate hues – intermediate locations. A, B) Continuous motion (constant unperturbed planar flow motion, left column) may result (A) in a *stable perception* of illusory rotation or (B) in a *spontaneous switch*. In the latter case the illusory sphere reverses its direction of rotation (right column), while the individual dots alter both their illusory depth and direction of rotation (middle column). See also Video S1. C, D) Forced ambiguous switch (planar flow reverses its direction of motion, left column) may result (C) in an *illusory motion reversal*: illusory rotation of an illusory sphere is reversed (right column), illusory rotation of individual dots is also reversed, but depth remains constant (middle column). See also Video S2. D) Alternatively, illusory motion may remain constant (*constant illusory motion* outcome): here illusory rotation of both the illusory shape and of the individual dots remains constant, but the illusory depth of the dots is altered (middle and right columns). See also Video S3.

While we are used to our perception being stable, this is not always the case. Certain displays contain ambiguous information which can be interpreted in a number of ways. In response, the visual system does not settle for a single interpretation and instead switches between alternatives in a semi-stochastic manner (for reviews see [1], [2]). Classic examples of such multi-stable displays include binocular rivalry [3], [4], Necker cube [5], dots' quartet [6] and structure-from-motion, also known as “kinetic-depth effect” [7], [8]. In structure-from-motion a two dimensional planar flow (Figure 1A, left column) is consistent with smooth rotation in depth. Despite the lack of true depth information, our visual system adds an inferred illusory depth as well as illusory motion-in-depth for each individual dot (Figure 1A, middle column) and uses the resultant velocity field to interpolate a rotating sphere (Figure 1A, right column). In the absence of disparity, luminance or size cues inferred illusory depth is ambiguous and, during continuous viewing the illusory rotation of the inferred sphere spontaneously switches between alternative states, along with illusory motion and depth of individual dots (Figure 1B, see also Video S1).

What makes structure-from-motion particularly interesting is the relationship between dots and the illusory sphere (or some other shape) which they define. Dots serve as the evidence for the sphere. Their spatial, luminance, size or disparity distributions determine the properties and number of the inferred illusory objects [9]–[16]. In other multi-stable displays, like the dots quartet [6], the dots themselves are objects of perception. For the structure-from-motion it is the interpolated illusory object/surface which dominates the perception. For example, even fairly large gaps in the distribution of dots may be hard to detect, if they interpolate to the same smooth illusory surface [17]. The fate of individual dots may be completely dissociated from the interpolated illusory sphere. For example limiting the dots' lifetime has virtually no effect on stability of an ambiguously rotating illusory sphere [11]. Even more surprisingly, reversal of the entire planar flow motion may be completely missed by an observer with illusory object continuing to rotate in the same direction [17], [10], [18].

The two possible outcomes for the latter manipulation are presented on Figure 1CD. Because the same sequence of physical events leads to two different interpretations, we will refer to it as the *forced ambiguous switch* (FAS). An inversion of a planar flow motion (Figure 1CD, left column) creates a conflict, as the planar motion is incompatible with an original combination of the inferred illusory depth and motion of individual dots. There are two alternative interpretations of this change. First, individual dots may reverse their illusory rotation, but keep the illusory depth (Figure 1C, *illusory motion reversal* outcome, see also Video S2). The illusory sphere has to follow and also reverse its direction of illusory rotation (Figure 1C, right column). Alternatively, direction of illusory rotation for both individual dots and illusory sphere may remain constant, but instead dots jump in depth to an opposing hemisphere (Figure 1D, *constant illusory motion* outcome, see also Video S3).

Although outcome of the FAS is ambiguous, certain display configurations strongly bias perception towards one of them. Limiting dots' lifetime almost completely abolishes illusory motion reversals [17]. Conversely, if an illusory object is not symmetric in depth at the time of FAS (due to its shape or polar projection) observers virtually always report a reversal of illusory motion [18], [10]. However in general, same sequence of physical events may result in illusory motion reversals in some trials and constant illusory motion in others [19], [20], [18].

Here we have explored how different forms of attention and stimulus properties influence outcome of FAS. The initial evidence from the spatial attention experiments, as well as from the parametric display manipulation, suggested that tracking of individual dots may be critical for illusory motion reversals. However, experiments with volitional control and feature/object attention failed to reveal the dependence on covert tracking. Instead, it appears that availability of spatial attention and reduced crowding allows subgroups of dots to form “features” on the surface of illusory sphere, “distorting” it and making it non-uniform. If the illusory object is not depth symmetric at the time of FAS, its own illusory depth cannot stay the same when the illusory depth of individual dots reverses. As we showed earlier [10] asymmetric shapes bias the visual system towards an alternative interpretation: a reversal of illusory motion.

## Results

### Experiment 1: Effect of spatial attention

**Figure 2:**
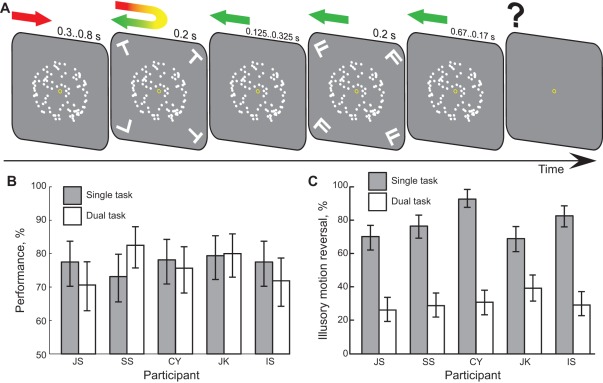
Effect of availability of spatial attention on the outcome of FAS. A) Experimental procedure (see Methods for details). A set of four letters was presented around the time of FAS, “bracketing” it by ±100 ms and was followed shortly by a mask. The response was collected after the stimulus presentation. B) Performance for the letter task in a single task (observers responded on letter task only) and dual task conditions. C) For all observers (near) absence of attention resulted in a highly significant drop of illusory motion reversal reports.

In our first experiment we examined the effect of spatial attention availability on the outcome of FAS. To this end, we paired structure-from-motion display with an attention demanding task. Assuming that attention is a single integrated resource, this should ensure near absence of attention on planar flow at the time of FAS [21], [22]. The secondary task consisted of four letters presented for 200 ms, bracketing time of FAS by 100 ms (Figure 2A). Observers reported 1) whether all letters in the set had the same identity (single task, letter task only condition); 2) whether initial and final direction of illusory rotation were identical (single task, full attention condition); 3) first on letter task, then on illusory rotation (dual task, poor attention condition).

For the letter task, the observers' performance was not significantly different between conditions: 77±1% for the single task and 76±2% for the dual task–paired sample t-test T4 = 0.277, p = 0.80 (see Figure 2B). Given its high attention demand [21], we are confident that most of the attention was removed from the structure-from-motion stimulus at the time of the switch.

For all observers, the poor attention condition resulted in a dramatic and highly significant drop in reversals of illusory motion (Figure 2B). The average decrease across five observers was 47±12% (T4 = 8.82, p = 0.001, paired sample t-test). However, near absence of attention did not completely abolish the motion change outcome, as all observers still reported it in ∼20–30% of the trials.

### Experiment 2: Eye movements during forced ambiguous switch

Results of the first experiment showed a strong effect of attention when it was manipulated in an all-or-nothing manner. We wondered whether minute fluctuations in attentional state [23]–[25] would have a measurable effect on the outcome of FAS. To estimate observers' attentional state, we recorded eye movements. Specifically, we monitored microsaccades: involuntary miniature ballistic eye movements observed during fixation intervals [26]. Frequency of their occurrence has been associated with higher attentional or perceptual demand of the task [27]–[29]. Accordingly, **lower** microsaccade rate should be associated with illusory motion reversal outcome. However, eye movements have been shown to facilitate spontaneous switches during continuous viewing of multi-stable displays [30], [31], [11]. This gives us an opposite prediction, namely that illusory motion reversals should be correlated with **higher** microsaccade rate in the interval immediately preceding FAS.

The experimental procedure was similar to that of Experiment 1, however FAS always occurred at 0.5 s following stimulus onset. A fixed schedule was used to ensure even statistical power across different time bins.

**Figure 3:**
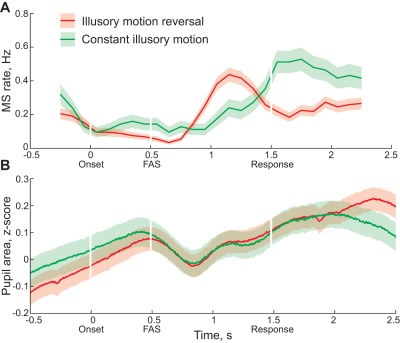
Microsaccades and pupil changes during FAS. A) Microsaccade rate, Hz. Mean ± standard error. Sliding time bin, width 0.5 seconds, step 0.1 seconds. B) Pupil area, z-score. Mean ± standard error.

Analysis of eye movements showed that illusory motion reversals were associated with lower microsaccade rate in the interval *preceding* FAS (Figure 3A). An average microsaccade rate during the first 0.5 seconds of presentation was 0.09±0.02 Hz for *illusory motion reversal* trials and 0.014±0.03 Hz for trials with *constant illusory motion* (time bin [0..0.5] seconds, paired sample t-test, T2 = 22, p = 0.002). This is consistent with our first hypothesis and suggests that higher attentional concentration may manifest itself in both lower microsaccade rate and tendency of observers to perceive an illusory motion reversal.

Alternatively, it is possible that microsaccades occurring around the time of FAS may simply mask motion transients generated by planar flow reversal. In this case a higher microsaccade rate trivially results in fewer illusory motion reversals, regardless of observers' attentional state. However, microsaccade rate was very low in both outcomes. Of all trials without illusory motion reversal, less than 4% contained a microsaccade around the time of the FAS (time bin [0.4..0.6] seconds, MS rate 0.2 Hz). Accordingly, microsaccadic suppression alone cannot explain the difference between perceived outcomes.

We also tested to see if the pattern of eye movements before FAS was different between outcomes. We did not find a significant difference in the amplitude of microsaccades prior to FAS (time bin [0..0.5] seconds, paired sample t-test, T2 = 0.07, p = 0.95). However, we found that smooth pursuit movements were slightly but significantly more likely for trials with illusory motion reversals (0.03±0.002 Hz) than during the constant illusory motion (0.025±0.001 Hz, paired sample t-test, T2 = 4.37, p = 0.0486). This suggests that the observers may have been tracking individual dots more often during illusory motion reversal trials. However, very low rates suggest that overt tracking does not play a major role in determining the trial outcome.

Finally, we analysed changes in pupil dilation, as pupil dilation is associated with a spontaneous switch and has been used to predict its occurrence [32] (but see [33]). Here we found no difference between conditions for time-bins either before FAS (time bin [0..0.5] seconds, paired sample t-test, T2 = −0.91, p = 0.46) or after FAS (time bin [0.5..1] seconds, paired sample t-test, T2 = −0.79, p = 0.52), see Figure 3B.

### Experiment 3: Effect of stimulus parameters

Our first two experiments established that availability of spatial attention facilitates illusory motion reversals. There are several possible mechanisms which together or separately may explain this effect.

First, attention is likely to act as an “effective contrast” (see [34] for a recent comprehensive review on visual attention), making motion transient stronger. This will make evidence for the change stronger and may facilitate reversals of illusory motion. For structure-from-motion displays, higher numbers of dots as well as faster rotation have been suggested to elevate neural responses [11]. Here we use them in order to try to replicate the “effective contrast” effect.

Additionally, attention may increase spatial resolution [35] and allow for easier tracking of individual dots. The latter may not be a necessary condition for illusory motion reversals (as near absence of attention in Experiment 1 would prevent it) but may be used by observers when attention is available (hence significantly more frequent smooth pursuit eye movements in Experiment 2). Interestingly, this would give us an opposite prediction since higher density would counteract any increase in spatial resolution due to attention. Similarly, both higher dot density and higher velocities should make tracking more problematic [36] and lead to fewer illusory motion reversals.

**Figure 4:**
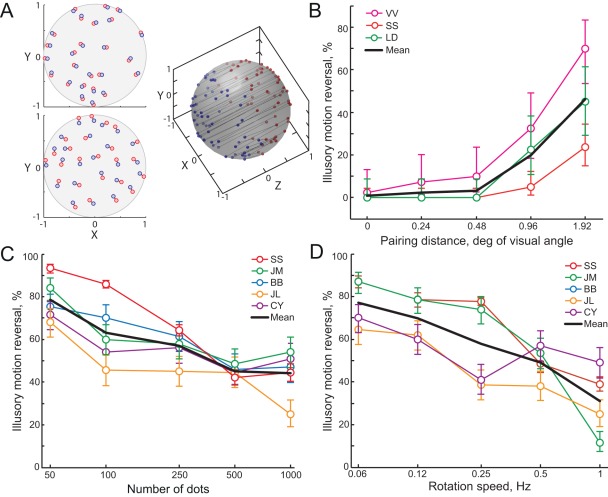
Effect of stimulus parameters on the outcome of FAS. A) Schematic representation of pairing of dots from “front” and “back” surface. Dots were placed so as to achieve a predefined distance on the screen (XY plane) at the moment of the forced ambiguous switch. Colour denotes surface ownership. Top: pairing distance of 0.24°. Bottom: pairing distance of 0.48°. B, C, D) Effect of the pairing distance (B), number of dots (C), and rotation speed (D) on the outcome of FAS. Mean ± standard error.

To better compare these alternatives, we varied not only the number of dots and speed of rotation of the illusory sphere, but also the pairing distance between dots belonging to the “front” and “back” surfaces at the time of FAS (Figure 4A). This keeps the number of dots constant and so the level of neural responses should also remain constant. However, smaller pairing distance would counteract enhanced spatial resolution and make tracking of individual dots through change more difficult. Note that zero pairing distance means complete occlusions of “back” surface dots by “front” surface ones, leading to the absence of motion transient. In this special case there is no physical motion reversal and observers tend to report no perceived changes [10].

Experimental procedure was similar to that of Experiment 2, except for randomized timing of FAS.

The results of Experiment 3 are presented in Figure 4BCD. In all cases stimulus parameters that lead to less crowding and/or easier tracking (i.e. bigger pairing distance, lower number of dots and lower rotation speed) result in more frequent illusory motion reversals. The results from an independent samples ANOVA shows a significant main effect for each manipulation: F4,10 = 7.15, p = 0.006 (pairing distance as an independent factor); F4,15 = 5.59, p = 0.0058 (number of dots as an independent factor); and F4,15 = 5.61, p = 0.0058 (rotation speed as an independent factor). These results are consistent with the hypothesis that illusory motion reversals are associated with trials when observers track individual dots.

The effect that neural adaptation has on our results also needs to be considered. It is clear that it must exert some effect: build-up of neural adaptation is one of the presumed causes of spontaneous switches [37]–[40] and FAS gives a suppressed percept an extra chance to overcome its competitor. We have re-analysed data from the rotation speed manipulation condition to see whether longer pre-FAS interval would lead to more frequent reversals of illusory motion. We have divided all trials into “early” (FAS occurred 0.5–0.75 seconds after the stimulus onset) and “late” (FAS occurred 0.75–1.0 seconds after the stimulus onset). On average, longer pre-FAS interval increased probability of illusory motion reversal by 3±1.9%. While this effect is significant (F1,12 = 5.87, p = 0.032, ANOVA with the onset time, the rotation speed and the observer identity as independent factors), its magnitude is fairly small making neural fatigue only a minor factor in FAS, at least for brief trial durations used in this study. We also looked at the interaction between rotation speed and duration of pre-FAS interval: Brower and van Ee [11] suggested that higher speeds may result in elevated neural responses, which in turn would lead to quicker build-up of adaptation. We do not find a significant interaction effect (F4,12 = 3.03, p = 0.061). However, fairly brief presentation times may have precluded us from detecting the effect.

### Experiment 4. Effect of volitional control

Results of previous experiments suggest that tracking of individual dots through FAS may facilitate illusory motion reversals: all conditions which are associated with easier tracking result in more frequent illusory motion reversals. Earlier reports are also consistent with this hypothesis. For example [19] replaced dots with gabor patches [19]. Use of orthogonal orientations for gabors belonging to “front” and “back” surfaces, which makes tracking easier, resulted in more frequent illusory motion reversals. Even though in all our experiments observers were instructed to passively observe the display, they may have employed such covert tracking to enhance their perception of motion.

To control for this possibility, we repeated the pairing dot condition of Experiment 3 but used explicit instructions regarding volitional control over the illusory sphere. Observers were prompted to 1) passively observe the stimulus (*passive* condition, a replication of Experiment 3); 2) attempt to force a reversal of illusory motion (*switch* condition), or 3) attempt to prevent any illusory motion reversals (*hold* condition). To examine whether the observers' strategy relied on the instance of FAS we included catch trials (25% of total trials), which contained no physical motion reversals.

**Figure 5:**
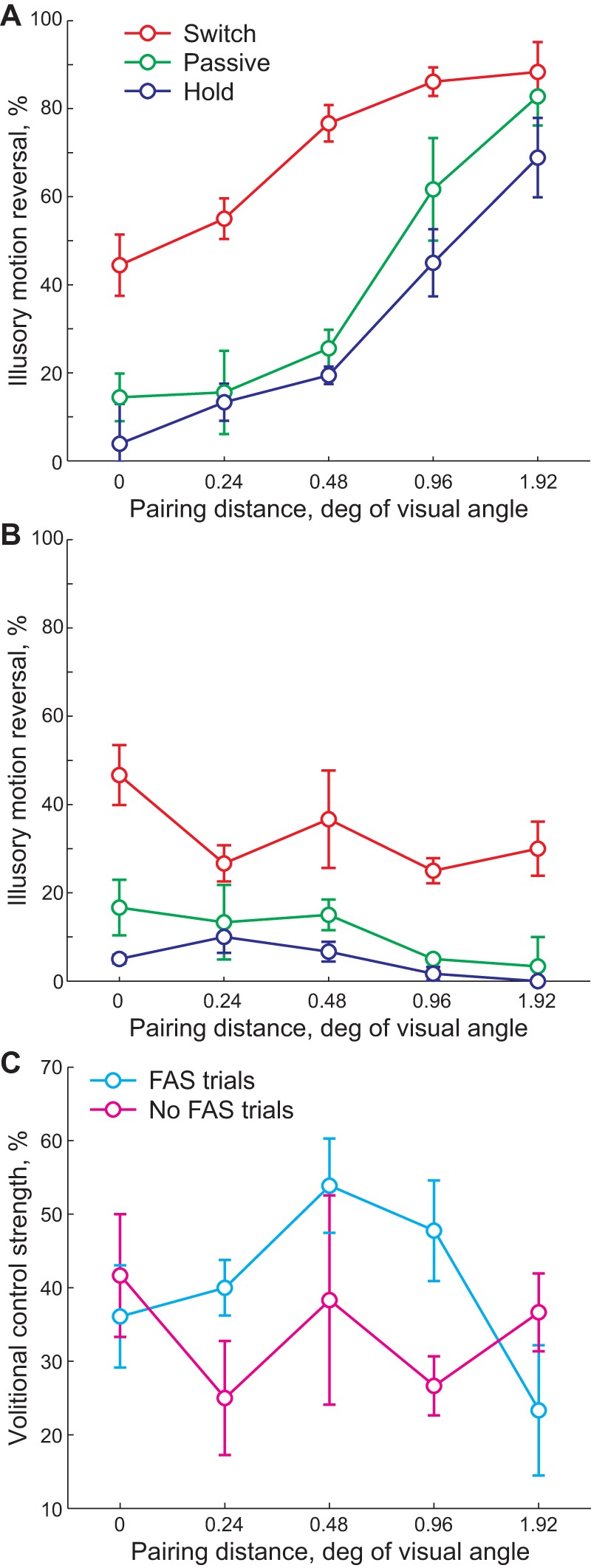
Volitional control over ambiguous illusory sphere during trials with (A) and without (B) forced ambiguous switch. Mean ± standard error. C) Strength of the volitional control defined as a difference in the percentage of illusory motion reversals between Switch and Hold conditions (see text for details).

Consistent with the results of Experiment 3, pairing distance at the time of FAS has a strong effect on the probability of illusory motion reversal (Figure 5A, F4,45 = 7.54, p = 0.0001; ANOVA with pairing distance and instructions as independent factors). We find that observers have a large amount of control over the illusory motion: more illusory motion reversals are reported for the *switch* condition (Figure 5A, F2,45 = 17.83, p<0.0001). However, there is no interaction between these two factors (F8,45 = 0.39, p = 0.92), suggesting that observers do not rely on FAS for their volitional control.

To further examine whether observers used FAS to control illusory motion, we compared the strength of the volitional control for trials with and without FAS (Figure 5B). We defined strength of volitional control as the difference in number of illusory motion reversals reported during *switch* and *hold* condition: 
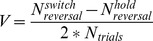
 (see Figure 5C). We find that strength of volitional control **does not** depend on either pairing distance (F4,30 = 0.39, p = 0.82; ANOVA with pairing distance and presence of FAS as independent factors), presence of FAS (F1,30 = 0.53, p = 0.47) or the interaction between these two factors (F4,30 = 0.56, p = 0.69). A paired sample t-test for individual pairing distances also failed to show any significance (all p>0.19). This reinforces the notion that FAS plays no part in volitional control, suggesting that observers are targeting spontaneous switches, as in the case of continuous presentation and unperturbed motion [11], [41].

### Experiment 5. Effect of attentional focus

The results of Experiment 4 showed that observers did not specifically target physical events of FAS in order to exert volitional control over illusory motion. Perhaps they did not realize that attending to dots could help them to induce more illusory motion reversals, or that ignoring them and focusing on an illusory sphere instead would make perception more stable? To test how feature or object attention would influence the outcome of FAS we repeated Experiment 4 but changed the instructions. Now observers had to either 1) passively observe the stimulus (*passive* condition, identical to that of Experiments 3 and 4); 2) focus attention on the illusory rotation of individual dots, ignoring the illusory sphere as a whole (*attend dots* condition), or 3) ignore the dots, focusing attention on the perception of the illusory sphere (*attend sphere* condition). In all conditions observers reported whether or not the illusory rotation (not the physical planar motion) has reversed during the trial.

**Figure 6:**
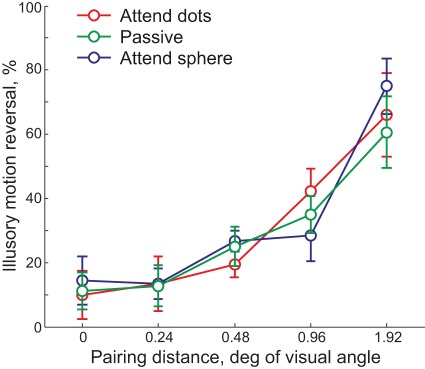
Effect of attentional focus on the outcome of FAS. Mean ± standard error.

The results of Experiment 5 are presented in Figure 6. Surprisingly, we find no significant difference between conditions. An independent samples ANOVA shows highly significant main effect of pairing (F4,45 = 14.84, p<0.001; ANOVA with the pairing distance and instructions as independent factors), but no main effect of instructions (F2,45 = 0.09, p = 0.92) or for interaction between the two (F8,45 = 0.012, p = 0.98). Such “blindness” to changes of the physical motion of individual dots may be surprising, but it is consistent with the surface interpolation hypothesis. Treue et al. [17] reported that even large irregularities in the spatial distribution of dots are hard to detect, if interpolated surface remains smooth. Experiments 1 and 2 showed that paying attention to the structure-from-motion strongly biases FAS towards illusory motion reversals. However, there appears to be no added effect of the covert tracking of individual dots' motion. This suggests that despite the instructions, observers were unable to focus their attention on dots. Instead the interpolated illusory sphere remained the primary focus of their attention, dominating the perception.

### Experiment 6. Effect of a presence/absence of a unique feature

Experiments 4 and 5 showed that the covert tracking of individual dots' motion has only a minor effect on illusory motion reversals (although it is still likely to account for at least some of them). This brings us back to the question of how attention availability and reduced crowding results in more frequent reversals of illusory motion.

**Figure 7:**
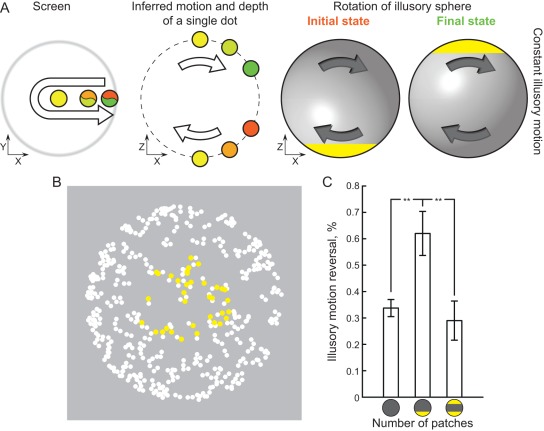
Effect of the colour patch. A) If the interpolated sphere is not symmetric relative to the zero depth plain at the time of FAS (e.g. as in a single patch condition), the *constant illusory motion reversal* outcome results in depth inversion for both individual dots (middle column) and the interpolated illusory sphere (right column). Compare to Figure 1D. B) Example sphere with a single patch, snapshot taken at the time of FAS. C) Presence of a single colour patch results in significantly more frequent illusory motion reversals.

It is possible that when individual dots are more distinct, they form a unique “feature” on the surface of the interpolated sphere, “distorting” it and making it non-uniform. This dramatically changes the perceived outcome of FAS with respect to the fate of the illusory sphere. It may no longer remain unchanged when illusory motion remains constant. As the dots which form the “feature” change their depth, the sphere also has to follow and change its own depth order (Figure 7A). Such inversion of the depth of the object is highly unlikely from an ecological point of view and the visual system typically opts for a more plausible change in illusory motion [10]. Indeed, when an illusory object is asymmetric in depth at the time of FAS due to its shape or a non-orthographic projection, illusory motion always reverses [18], [10]. Studies which used continuous motion showed that spontaneous switches tend to occur when the salient feature, produced with colour or via distortions in spatial distribution, is approximately symmetric in depth [10], [11].

This gives us a strong prediction: if a salient feature does not have a mirror counterpart at the time of FAS, it should strongly bias perception towards reversals of an illusory sphere. To test this we used three conditions: 1) uniform sphere with no distinct feature; 2) sphere with a single yellow patch (Figure 7B); 3) sphere with two symmetric yellow patches. We used a higher number of dots in planar motion flow (200) in order to make the appearance of the sphere more uniform. A patch was produces by colouring dots yellow within radius of 2.5°. Patches were placed so that they were centred at fixation at the time of FAS. Accordingly, when two colour patches were present they directly overlapped each other at the time of FAS. This means that sphere was symmetric relative to the zero depth plain at the time of the FAS in the first and third conditions, but not in the second one.

As can be seen in Figure 7C the results fully bear out our prediction. A pairwise t-test comparison shows a highly significant difference between uniform sphere and single patch condition (T38 = −3.24, p = 0.0025) and single and dual patch conditions (T38 = 3.52, p = 0.001), but not between uniform sphere and dual patch (T38 = 0.6, p = 0.55, significance level after Bonferroni correction for multiple comparisons was α = 0.017). A strong effect of the colour patch clearly supports our hypothesis that the grouping of dots into features distorts sphere uniformity and facilitates illusory motion reversals.

## Discussion

In the course of six experiments we have examined how attention and stimulus parameters influence outcome of the forced ambiguous switch. We found that poor attention on an ambiguously rotating sphere, either due to competing attention-demanding tasks or due to natural fluctuations in attentional state, results in fewer reversals of illusory motion. For stimulus parameters we found that illusory motion reversals become more frequent if the sphere is comprised of fewer dots, is moving slower, or if individual dots are further away from their opposing hemisphere counterpart at the time of FAS. While these results are compatible with illusory motion reversals being facilitated by covert tracking individual dots, additional experiments on volitional control and feature/object attention did not support this hypothesis. Although observers showed a large degree of voluntary control over the ambiguously rotating sphere, it was not in any way linked to FAS. Moreover, prompting the observer to selectively pay attention to the dots or the illusory sphere had no measurable effect on the outcome of FAS. Overall, top-down attention has a strong effect: even small fluctuations in the attentional state influence outcome of FAS. However, it clearly operates at the level of the interpolated illusory sphere with only limited and/or an indirect access to the representations of individual dots.

The fact that the tracking of individual dots is of little consequence to reversals of the illusory motion is startling. It is even more surprising if one considers earlier findings [17], that limited-lifetime-dots completely abolish reversals of illusory motion, as these suggest that the ability to track dots is crucial for such reversals. Yet, results of Experiments 4 and 5 argue strongly against this conclusion. Another experiment in the same study [17] confirmed the limited importance of tracking: when observers tracked a single salient dot, they reported a vivid displacement of this dot in depth, rather than a reversal of illusory motion of an illusory cylinder. Taken together, these observations argue that the tracking of individual dots does not directly cause reversals of illusory motion, even though it may be contributing in some cases.

It appears that the second prerequisite for the illusory motion reversal, along with attention availability, is an asymmetry of the inferred shape. Such asymmetry can emerge if some dots are grouped together and form a “feature” on the surface of the illusory sphere (in an extreme case, e.g. with complete symmetry, such grouping can even result in perception of multiple surfaces [9]). Consistent with this hypothesis, the presence of an asymmetric colour patch results in a highly significant increase in reports of illusory motion reversals. This also helps us explain the results of the stimulus parameters manipulation. Denser displays would make an interpolated surface more uniform, leading to the observed decrease in reported illusory motion reversals. Smaller pairing distance would be likely to work in two ways. First, it would make dots belonging to “front” and “back” surface more confusable, reducing visibility of individual grouped “features”. Second, smaller pairing would result in an interpolated sphere being more symmetric, with respect to the zero depth plane at the time of FAS.

Asymmetry relative to the zero depth plane at the time of FAS is critical because it changes possible outcomes with respect to the illusory sphere. If the illusory sphere is interpolated as uniform, following a depth switch of *individual dots*, these dots interpolate to the same surface (see Figure 1D). Accordingly, the illusory sphere remains constant and, from the top level representation point of view, the world remains stable. However when a group of dots, which formed a “feature” on the interpolated surface, changes its depth, the illusory sphere has to follow (this is illustrated on Figure 7A).

**Figure 8:**
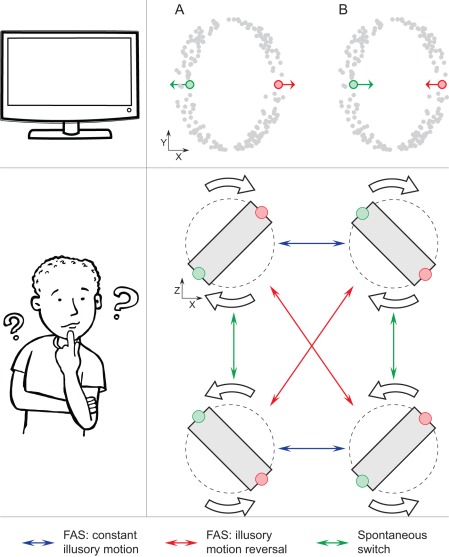
Illusory motion and depth of a rotating band. Illustrated are the instants just before and after FAS, with identical spatial distributions but opposite directions of motion. Top row: frontal views with two highlighted example dots (red and green). Middle and bottom rows: top views of the interpolated shapes, resulting from different combinations of illusory motion and illusory depth, again with two highlighted example dots (red and green). The arrows depict spontaneous reversals (green), reversals of illusory depth only (blue, constant illusory motion outcome of FAS), and reversals of illusory rotation only (red, illusory motion reversal outcome of FAS).

This is best illustrated with an object that is neither rotationally symmetric nor depth-symmetric at the time of FAS, as illustrated in Figure 8. The top row depicts two static snapshots of the illusory band (as used in [10]) that are not depth-symmetric (front view). While the spatial distribution of dots is identical, their planar motions are opposite (compare red and green example dots). The two bottom rows depict the possible combinations of illusory depth and motion in the interpolated shape (top view), as illustrated by the red and green example dots. During a spontaneous reversal (Figure 8, green arrows), the illusory depth of the entire band and of individual dots are linked and must reverse together (or not). The same linkage characterizes the reversal of illusory depth (Figure 8, blue arrows) or of illusory rotation (Figure 8, red arrows) during FAS.

As we have recently reported, even when dealing with illusory states generated by ambiguous displays, the visual system takes into account the ecological validity of transformations between them [10]. If a transition to the alternative percept is deemed implausible, spontaneous switches do not occur. In case of FAS, a change in (illusory) motion is treated as a far more likely event than the inversion of an (illusory) object in depth, and the visual system typically prefers the former. This is particularly easy to see for non-uniform shapes (as we have illustrated above) or using polar projection. As object shape is asymmetric in depth at the time of FAS, inversions of the object are very rarely perceived and illusory motion typically reverses instead [10], [18], [42].

It is likely that attention interacts with the surface interpolation mechanism. Both an enhanced spatial resolution [35] as well as grouping, which is facilitated by attention [43]–[48], are likely to amplify small differences and/or regularities present in the spatial distribution of the dots, making resultant illusory sphere less uniform and less symmetric. Accordingly, attention mainly affects representation of the inferred illusory sphere, rather than being directly involved in interpretation of the events during FAS.

The feature asymmetry hypothesis also bodes well with prior reports on FAS. For example use of orthogonally oriented gabors facilitates reversals of illusory motion [19]: as vertically oriented gabors are not easily “interchangeable” with the horizontally oriented ones, the depth inversion is ruled out in favour of illusory motion reversal. Conversely, structure-from-motion displays with limited life time dots almost never produce an illusory motion reversal [17]. Here limited lifetime is likely to prevent formation of stable features making an interpolated surface very uniform and, thus, symmetric in depth.

Prior research showed importance of non-motion factors, like shape and symmetry, for the perception of structure-from-motion [9], [10], [42]. Here we extend these findings, showing that distributions of dots may distort a form of the interpolated sphere-from-motion. This is not surprising, given an overlap between brain areas involved in processing of structure-from-motion and 3D form from other cues like disparity, texture and shading [49], [50]. Neural correlates of such form asymmetry are likely to be found in the parietal region (e.g. V3A, V7, various areas in intraparietal sulcus) [49], [50] and the LOC [9].

The same mechanism is likely to restrict switching during continuous (unperturbed) stimulus presentation of structure-from-motion. Brower and van Ee [11] showed that if a patch is formed on the surface of the sphere using disturbances in spatial distribution of dots, spontaneous switches tend to occur when this “patch” is approximately symmetric in depth. We have recently reported that a colour stripe on the surface of the sphere produces a similar effect and constrains spontaneous switches to specific angles of rotation almost as effectively as a stripe-shaped object is capable of by itself [10]. In Experiment 3, when we systematically varied the number of dots and their speed, we were able to make a direct comparison with work of Brower and van Ee on the spontaneous switch [11]. At face value our results are directly opposite: Brower and van Ee found that having a higher dot number and faster speeds results in a reduction of perceptual stability (more reversals of illusory motion are observed), whereas our results showed an increase in perceptual stability (fewer reversals of illusory motion are reported). However what matters is the link between the fate of individual dots and that of the interpolated sphere: in both cases more numerous dots and faster velocities facilitate the dissociation between the two. For the Brower and van Ee paradigm this means that illusory motion reversals occur despite continuous physical motion, for FAS – that illusory sphere remains stable despite reversals of the physical motion.

To summarize, our results lend further support to the hypothesis of surface interpolation. They show that even large changes to the physical motion of individual dots can be tolerated, as long as the interpolated illusory object as a whole remains unchanged. However, changes in physical motion may lead to the reversal of the illusory motion if the interpolated object is not depth symmetric. Shape asymmetry can emerge if a subset of dots is grouped together by the visual system to form an asymmetric feature. This process would seem to be strongly facilitated by attention.

## Materials and Methods

### Observers

Procedures were approved by the medical ethics board of the Otto-von-Guericke Universität, Magdeburg: “Ethik-komission der Otto-von-Guericke-Universität an der Medizinischen Fakultät”. All participants had normal or corrected-to-normal vision. Apart from the authors, observers were naïve to the purpose of experiments and were paid for their participation. Details on number of participants for each experiment are summarized in Table 1.

**Table 1:** Summary on observers participating in the study.

Experiment	Observers
1	Six observers (all females), including the first author. Data from one participant in the first experiment was not used in analysis, as she could not allocate attention properly (there was a significant drop in letter task performance under dual task condition, paired t-test p<0.045).
2	Three observers (all females), including the first author.
3	Seven observers (five females), including the first and third authors.
4,5,6	Four observers (two females).

### Apparatus

Stimuli were generated in Matlab, using the Psychophysics Toolbox [51] and displayed on a CRT screen (Iiyama VisionMaster Pro 514, iiyama.com) with a spatial resolution of 1600×1200 pixels and refresh rate of 100 Hz. The viewing distance was 73 cm, so that each pixel subtended approximately 0.019°. In all experiments background luminance was kept at 36 cd/m2 and environment luminance at 80 cd/m2.

Eye movements were recorded binocularly using a desktop mounted Eyelink 2000 eye tracker (SR Research, sr-research.com) using nine point calibration with acquisition rate of 1000 Hz. Microsaccades were extracted using an automated procedure as described in [52].

### Depth-from-motion stimulus

Planar motion flow was used to create an appearance of a three-dimensional rotating sphere, presented at fixation [7], [8]. The diameter of single dot was 0.057°, luminance–110 cd/m^2^, dots were semi-transparent to exclude any possible occlusion effects. Sphere speed of rotation was constant at 0.2 Hz. Sphere radius was 5.7° for experiment 1, 9.6° for experiments 2–6.

The number of dots which comprised the sphere varied between experiments and individual observers:

- Experiment 1: 250 dots.
- Experiment 2: 50..1000 dots. Large variability is due to very different settings required to achieve a 50/50 balance between outcomes of FAS for individual observers.
- Experiment 3a, 4 and 5: 30..50 dots.
- Experiment 3b, effect of global density: 50..1000 dots.
- Experiment 3c, effect of rotation speed: 30..50 dots.
- Experiment 6: 200 dots.

### Letter discrimination task

A set of four letters (Ts and/or Ls) was briefly (200 ms) presented around the structure-from-motion stimulus bracketing the time of the dots' motion reversal by ±100 ms, see Figure 2A. They were followed by a mask (letters F, 200 ms). SOA range ([125..325] ms) was selected individually for each observer based on their single task results to achieve ∼75% performance. Each letter subtended 0.96° of visual angle and were 5.4° away from the fixation point in 45°, 135°, 225°, and 315° directions. Observers reported whether all letters were identical or not by pressing *F* (all identical) or *J* (one odd letter) with the *left hand*.

### Experiment 1. Effect of spatial attention

Depth-from-motion stimulus was presented for 1.5 s accompanied by the letter discrimination task (see **Figure **2**A** and **Letter discrimination task** above for timeline details). Time of forced ambiguous switch (FAS, reversal of planar motion flow, see Introduction section for details) was drawn from a uniform random distribution between 0.5 s and 1 s. Observers reported on 1) letter task alone: left hand, preliminary single task session to establish individual SOAs for ∼75% performance; 2) whether the initial and final directions of rotation of the ambiguous sphere were identical or not: right hand, cursor keys, single task, full attention condition; 3) first on the letter task (left hand), then on the illusory rotation (right hand), dual task, poor attention condition. Five observers participated in the experiment.

### Experiment 2. Eye movements during forced ambiguous switch

The depth-from-motion stimulus was presented for 1.5 s with FAS occurring at 0.5 s after the stimulus onset. Timing of FAS was fixed to ensure an equal number of samples across all time bins during later analysis. The dot number in the sphere was varied across blocks to ensure a ∼50/50 balance of forced ambiguous switch outcomes. The number of dots was decreased whenever the percentage of motion change reports was above 60% and increased when it was below 40%. The following numbers were used: 50, 75, 100, 150, 200, 250, 300, 500, 750 and 1000. The large variability is due to very different settings required to achieve a 50/50 balance between outcomes of FAS for individual observers. Observer reported whether they had perceived a motion change by pressing cursor keys using the right hand. Three observers participated in the experiment.

### Experiment 3. Effect of stimulus parameters on the outcome of the forced ambiguous switch

Depth-from-motion stimulus was presented for 1.5 s. Time of forced ambiguous switch was drawn from a uniform random distribution between 0.5 s and 1 s. The observer reported whether they had perceived a motion switch (initial and final direction of rotation of illusory shape differed) by pressing the cursor keys using their right hand (*Left* – constant illusory motion, *Right* – reversal of illusory motion). Seven observers participated in the experiment.

In Experiment 3a (pairing distance) the dots that make up the rotating sphere were placed in such a manner as to be at a fixed distance in the XY (screen) plane from their opposing hemisphere counterpart at the time of FAS (see Figure 4A). This distance was systematically varied between the blocks (0°, 0.24°, 0.48°, 0.96° and 1.92° of visual angle). In Experiment 3b we systematically varied the number of dots (50, 100, 250, 500 and 1000). In Experiment 3c speed of rotation was systematically varied between blocks (0.06, 0.12, 0.25, 0.5 and 1.0 Hz).

### Experiment 4. Effect of volitional control

The procedure was identical to Experiment 3a with additional instructions for the volitional control. 25% of trials did not have FAS (catch trials). Observers were instructed to 1) passively observe the stimulus (*Passive* condition), 2) attempt to induce a reversal of illusory rotation (*Switch* condition), 3) attempt to hold direction of illusory rotation constant (*Hold* condition). Observers were instructed to maintain their fixation and the quality of their fixation was informally monitored. Four observers participated in the experiment.

### Experiment 5. Effect of attentional focus

The procedure was identical to Experiment 4 except for the given instructions. Observers were instructed to 1) passively observe the stimulus (*Passive* condition), 2) focus attention on the dots, ignoring the illusory sphere (*attend dots* condition), 3) ignore the dots, focusing attention on the illusory sphere (*attend sphere* condition). Observers were instructed to maintain their fixation and the quality of their fixation was informally monitored. Four observers participated in the experiment.

### Experiment 6. Effect of a presence/absence of a unique feature

The procedure was similar to the passive condition of Experiment 4 and 5. A higher number of dots (200) was used in order to make the appearance of the interpolated illusory sphere more uniform. The sphere was either uniformly coloured (*no patch* condition), had a colour patch only on one surface (*single patch* condition, see B), or had two symmetric colour patches (*two patches* condition). The patches were produced by colouring subset of the dots yellow. The patches had a radius of 2.5° and were placed in a set position so that they would always be in the position of fixation at the time of FAS. Four observers participated in the experiment.

## Supporting Information

Video S1 **Structure-from-motion display also referred to as kinetic-depth effect or depth-from-motion.** A planar flow is perceived as an illusory sphere rotating in depth. Due to an ambiguous illusory depth, the front surface can be perceived as moving left or right. Perception will spontaneously change between alternatives during continuous viewing (please ensure that movie is looped).
(MOV)

Video S2 **Forced ambiguous switch: large pairing distance favours illusory motion reversals.** For most observers the sphere appears to reverse its direction of the illusory rotation during the presentation. See text for further details.
(MOV)

Video S3 **Forced ambiguous switch: small pairing distance favours constant illusory motion.** For most observers the sphere appears to retain initial direction of the illusory rotation for the entire trial. The moment of the forced ambiguous switch may be perceived as a brief “hesitation” in the illusory motion. See text for further details.
(MOV)
